# Enrichment of genomic pathways based on differential DNA methylation profiles associated with chronic musculoskeletal pain in older adults: An exploratory study

**DOI:** 10.1177/1744806920966902

**Published:** 2020-10-18

**Authors:** Soamy Montesino-Goicolea, Puja Sinha, Zhiguang Huo, Asha Rani, Thomas C Foster, Yenisel Cruz-Almeida

**Affiliations:** 1 Pain Research & Intervention Center of Excellence, University of Florida, Gainesville, FL, USA; 2 Department of Community Dentistry & Behavioral Science, College of Dentistry, University of Florida, Gainesville, FL, USA; 3 Department of Neuroscience, College of Medicine, University of Florida, Gainesville, FL, USA; 4 Department of Biostatistics, College of Public Health & Health Professions and College of Medicine, University of Florida, Gainesville, FL, USA; 5 Genetics and Genomics Program, University of Florida, Gainesville, FL, USA; 6 Institute on Aging, University of Florida, Gainesville, FL, USA

**Keywords:** Chronic musculoskeletal pain, DNA methylation, gamma-aminobutyric acid, genomic pathways, immune signaling, older adult

## Abstract

Our study aimed to identify differentially methylated CpGs/regions and their enriched genomic pathways associated with underlying chronic musculoskeletal pain in older individuals. We recruited cognitively healthy older adults with (*n* = 20) and without (*n* = 9) self-reported musculoskeletal pain and collected DNA from peripheral blood that was analyzed using MethylationEPIC arrays. We identified 31,739 hypermethylated CpG and 10,811 hypomethylated CpG probes (*p*s ≤ 0.05). All CpG probes were clustered into 5966 regions, among which 600 regions were differentially methylated at *p* ≤ 0.05 level, including 294 hypermethylated regions and 306 hypomethylated regions (differentially methylated regions). Ingenuity pathway enrichment analysis revealed that the pain-related differentially methylated regions were enriched across multiple pathways. The top 10 canonical pathways were linked to cellular signaling processes related to immune responses (i.e. antigen presentation, programed cell death 1 receptor/PD-1 ligand 1, interleukin-4, OX40 signaling, T cell exhaustion, and apoptosis) and gamma-aminobutyric acid receptor signaling. Further, Weighted Gene Correlation Network Analysis revealed a comethylation network module in the pain group that was not preserved in the control group, where the hub gene was the cyclic adenosine monophosphate-dependent transcription factor ATF-2. Our preliminary findings provide new epigenetic insights into the role of aberrant immune signaling in musculoskeletal pain in older adults while further supporting involvement of dysfunctional GABAergic signaling mechanisms in chronic pain. Our findings need to be urgently replicated in larger cohorts as they may serve as a basis for developing and targeting future interventions.

## Introduction

Chronic pain prevalence increases with age leading to significant distress and disability.^1–5^ In particular, current interventions do not provide sufficient levels of pain relief in older individuals with musculoskeletal pain.^6,7^ Further, available pain treatments such as nonsteroidal anti-inflammatories are often accompanied by detrimental side effects that limit their long-term use in this vulnerable population. Although there is an increasing understanding of potential neurobiological mechanisms underlying musculoskeletal pain, mechanistic human studies are currently lacking, which may help identify potential therapeutic targets in the older population.

Many of the neurobiological contributors to chronic pain development and maintenance are also a by-product of long-lasting gene alterations along multiple levels of the neural axis. Epigenetic mechanisms are emerging as factors that impact gene expression and may account for the gene–environment interactions that are an inevitable part of the pain experience. To date, one of the most studied epigenetic modifications is DNA methylation. The best characterized DNA methylation process is the covalent addition of a methyl group to the 5th carbon of a cytosine residue followed by a guanine residue (i.e. CpG), leading to targeted gene transcriptional repression. On the other hand, DNA demethylation, or removal of the methyl group, leads to transcriptional activation. Although epigenetic modifications were originally thought to only program patterns of gene expression during cellular development and differentiation, a growing body of research suggests that these modifications may also occur in response to environmental exposures throughout the lifespan. Thus, these epigenetic modifications appear to significantly change gene regulation, neural plasticity, and subsequently behavior.^8^

DNA methylation has been implicated in the induction and maintenance of pain in animals and humans.^9–13^ Specifically, DNA methylation levels have been found to significantly differ between controls and individuals reporting low-back pain,^14^ neuropathic pain,^15–17^ and in women with fibromyalgia.^18^ However, to our knowledge, no study has examined epigenetic differences among older adults with and without musculoskeletal pain. Hence, in this exploratory study, we compared DNA methylation profiles between older adults with and without musculoskeletal pain during the past three months. We employed a pathway enrichment analysis to identify pathways enriched in DNA methylation differences, and we applied a Weighted Gene Correlation Network Analysis (WGCNA) to identify comethylation networks preserved in the chronic pain group compared to controls. We hypothesized that DNA methylation profiles would be significantly different between older adults with and without musculoskeletal pain.

## Materials and methods

Community-dwelling older individuals over 60 years of age who were native English speakers were recruited as part of a larger project at the University of Florida (Neuromodulatory Examination of Pain and Mobility Across the Lifespan (NEPAL)). Participants were recruited through posted fliers, newspaper ads, and word-of-mouth referrals. Potential participants were screened over the phone and again in person and were excluded if they reported (1) Alzheimer’s, Parkinson’s, or other neurological condition directly impacting the brain; (2) serious psychiatric conditions (e.g. schizophrenia, major depression, bipolar disorder); (3) blood pressure greater than 150/95 mm Hg, heart failure, or history of acute myocardial infarction; (4) systemic rheumatic diseases (i.e. rheumatoid arthritis, systemic lupus erythematosus, fibromyalgia); (5) chronic opioid use; (6) magnetic resonance imaging contraindications; (7) excessive anxiety regarding protocol procedures; (8) hospitalization within the preceding year for psychiatric illness; (9) HIV or AIDS; and (10) if they scored less or equal to 77 on the Modified Mini-Mental State Examination (3MS).^19^ All procedures were reviewed and approved by the University of Florida’s Institutional Review Board, and all participants provided verbal and written informed consent.

Participants came to the laboratory multiple times for the NEPAL study, and previous findings have been reported elsewhere.^20^ For the current exploratory investigation, pain assignment was performed during data analysis phase in a post hoc fashion. Participants were interviewed using a standardized pain history instrument regarding the presence of pain during the past three months across several body regions (i.e. head/face, neck, shoulders, arms, hands, chest, stomach, upper and lower back, leg, knees, and feet) using a validated body manikin.^21,22^ Individuals reporting pain on most days for more than three months on at least one body site were classified as having chronic pain. The pain reported by our older participants was considered mainly of nociceptive musculoskeletal origin, as we excluded putative neuropathic pain phenotypes where participants with a PainDETECT score of 12 or higher were tested for static and dynamic mechanical allodynia in the painful area. Individuals reporting any allodynia were subsequently excluded from the study. A subset of individuals (*n* = 29) underwent a blood draw in the antecubital fossa following standardized procedures. Venipuncture took place during the neuroimaging session of the NEPAL study. We used the R statistics package to calculate chi-square and *t*-tests to examine differences between pain groups with regards to demographics, and a *p* value less than 0.05 was considered statistically significant.

### Measurement of DNA methylation

Human blood samples were collected into 15 ml conical tubes treated with anticoagulant ethylenediaminetetraacetic acid (EDTA) in a random subset of participants of the NEPAL study. The samples were stored at –80°C until processing. To Isolate DNA, the frozen blood samples were thawed at 37°C to dissolve homogeneously. Whole blood samples (500 µl) were lysed in red blood cell (R.B.C) lysis buffer and centrifuged at 6000 r/min for 5 min at room temperature. The supernatant was discarded, and sodium EDTA solution was added to the pellet and vortex gently to remove RBC clumps. Homogenate was incubated at 50–55°C with Proteinase K and sodium dodecyl sulfate solution. Following incubation, equal volume of phenol was added, mixed, and centrifuged at 10,000 r/min for 10 min. Supernatant was transferred in a fresh tube, and equal volume of phenol-chloroform-isoamyl alcohol was added, mixed and centrifuged at the same r/min. Again, supernatant was transferred in a fresh tube, and equal volume of chloroform-isoamyl alcohol was added followed by centrifugation at same r/min conditions. Supernatant was transferred in a fresh tube, and 1/10th volume of 3 M sodium acetate along with 2 volumes of absolute alcohol was added. The precipitated DNA was washed with 70% ethanol by centrifugation at 10,000 r/min for 5 min. The pellet was air dried and dissolved in Tris-EDTA buffer. The quality of DNA samples was assessed using nanodrop (purity of 260/280 ratio from 1.8 to 2.0). The dissolved DNA was qubit quantified and visualized on agarose gel for quality check. Sodium bisulfite conversion of 500 ng–1 µg of input DNA using EZ DNA Methylation-Direct kit (Zymo Research) and EPIC methylation array was performed by Moffitt Cancer Center, Molecular Genomics Core 3011 Holly Dr. Tampa, FL 33612. The bisulfite-converted samples were hybridized in the Human Infinium Methylation EPIC BeadChip microarrays (Illumina Inc., Tampa, FL).

### DNA methylation data preprocessing

Methylation data preprocessing and quality control was performed by R package *minfi*.^23^ To be brief, sample-specific quality control was performed by *plotQC* function in the *minfi* package, and all our samples were of good quality (Figure S1). IlluminaHumanMethylationEPIC annotation files hg19 were used for mapping to the genome. Functional normalization was employed to perform between-array normalization and regress out variability explained by the control probes. Among all 865,859 CpG probes, we removed (1) 1150 probes with nonsignificant detection *p* value (*p* > 0.01) in more than 10% samples; (2) 30,064 probes which contain a single nucleotide polymorphism (SNP) either at the CpG interrogation or at the single nucleotide extension; and (3) 18,920 probes on the sex chromosome. Totally, 815,725 CpG probes remained in our final analysis.

### Identifying differentially methylated probes/differentially methylated regions associated with pain

To identify differentially methylated probes (DMPs) associated with pain, we employed the linear model, followed by the empirical Bayes moderated *t*-statistics test, which are implemented in the *limma* package.^24^ In this analysis, we adjusted for age, sex, and race as covariates. Since DNA methylations are highly correlated between adjacent CpG sites, pain-related CpGs can be clustered in genomic regions.^25^ Therefore, we also performed region-based analysis to identify differentially methylated regions (DMRs) associated with pain, using *bumphunter* method^26^ within R *minfi* package, which automatically performs genomic segmentation, creates CpG clusters, and identifies DMRs using a similar linear model approach. Statistical significance of a DMR was obtained by permutation test. Because of the small sample size and high correlation between CpG sites/regions in this exploratory study, we used raw *p* < 0.05 to determine statistical significance.

### Functional annotation and enrichment

To examine the potential functions of the identified DMPs/DMRs, we annotated them to genomic features, including promoters, exons, introns, and intergenetic regions, using the R package *GenomicFeatures*.^27^ Functional enrichment analysis was performed using Ingenuity Pathway Analysis.

### Comethylation networks

To examine whether CpG probes that are differentially methylated in relation to pain are comethylated, we conducted the WGCNA.^28^ This analysis included a total of 876 DMPs showing nominal associations (*p* < 0.001) with pain, after adjusting for age, sex, and race. Comethylated modules were constructed among subjects with pain. To explore whether the network structure of the comethylated module vary by pain status, we performed preservation analysis in the WGCNA. Hub genes within each comethylation module were detected using the *ARACNE* algorithm^29^ in the R package *minet*.^30^ Network visualization was done using *Cytoscape*.^31^

## Results

### Sample characteristics

Our older participants were cognitively intact, on average 71 years of age, mostly female, Caucasian, with no significantly reported depressive symptomatology. Participants reported musculoskeletal pain most commonly in the back and the knees, although they reported pain at multiple body sites. However, individuals reporting chronic pain had a significantly lower score on the 3MS compared to those without chronic pain (*p* = 0.003). Details on this subset of participants have been previously reported by our group^20^ and in Table 1.

**Table 1.** Characteristics of the study participants.

	Pain (*n* = 20)	No pain (*n* = 9)	*p* value
Age, mean (*SD*)	70.2 (5.1)	71.2 (8.0)	0.732
Males, *N* (%)	3 (15.0)	3 (33.3)	0.247
Race, *N* (%)			0.129
Non-Hispanic White	20 (100)	8 (88.9)	
Asian/Pacific Islander	0 (0)	1 (11.1)	
3MS, mean (*SD*)	99.5 (0.8)	96.8 (3.4)	0.003
CES-D, mean ± *SD* years	4.9 ± 3.8	8.1 ± 5.6	0.154
BMI, mean ± *SD*	25.9 ± 4.6	28.0 ± 5.4	0.329

3MS: Modified Mini-Mental State Examination; CES-D: Center for Epidemiologic Studies Depression Scale; BMI: body mass index.

### DMPs/DMRs associated with pain

**Figure 1.**
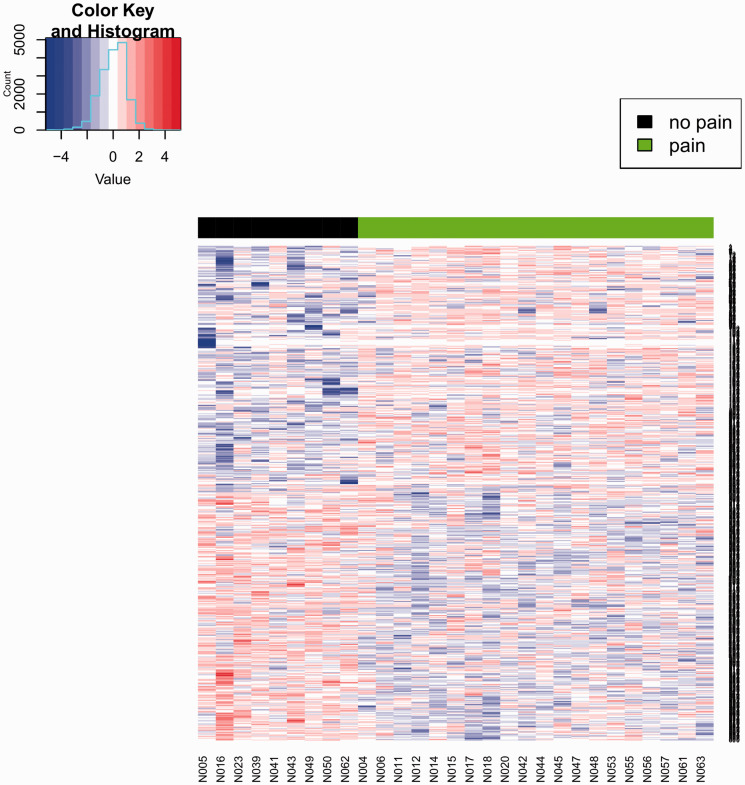
Heatmap visualization of all putative DMPs (*p* ≤ 0.05). The color key indicates the *z*-score of the methylation value. The colors red and blue indicate higher and lower methylation value, respectively. Black and green color bar on top of the heatmap indicates no-pain and pain groups, respectively.

At *p* ≤ 0.05 level, we identified 31,739 hypermethylated CpG probes and 10,811 hypomethylated CpG probes. The top 20 DMPs are shown in Table 2, and the full list is shown in Table S1. All CpG probes can be clustered into 5966 regions, among which 600 regions are differentially methylated at *p* ≤ 0.05 level, including 294 hypermethylated regions and 306 hypomethylated regions. The top 20 DMRs are shown in Table 3, and the full list is shown in Table S2. Figure 1 shows the heatmap visualization of the 600 putative DMRs.

**Table 2.** Top 20 differentially methylated probes.

CpG probe	Chr	Position (bps)	Genomic feature	Direction^a^	*p* value	Genes^b^
cg06492735	5	165,808,933	Intergenic	↑	1.07E-06	
cg07725536	13	93,211,487	Introns	↑	1.29E-06	GPC5
cg11131672	1	170,588,581	Intergenic	↑	4.59E-06	
cg20109472	20	49,613,314	Intergenic	↑	5.18E-06	
cg26752422	13	66,035,796	Intergenic	↑	8.22E-06	
cg04467406	19	42,210,465	Intergenic	↑	9.14E-06	CEACAM5
cg26220722	14	23,824,354	Intergenic	↑	1.13E-05	SLC22A17; EFS
cg09073308	5	65,808,717	Intergenic	↑	1.18E-05	
cg12267448	6	22,322,873	Intergenic	↑	1.21E-05	
cg15717719	2	24,150,218	Promoters	↓	1.36E-05	ATAD2B; UBXN2A
cg04240062	3	105,185,133	Introns	↑	1.46E-05	ALCAM
cg00651099	4	125,599,866	Exons	↑	1.55E-05	ANKRD50
cg00324205	15	94,911,890	Introns	↑	1.59E-05	MCTP2
cg13729903	12	107,169,414	Promoters	↑	2.01E-05	LOC100287944; RIC8B
cg03741931	11	8,204,883	Intergenic	↑	2.45E-05	
cg15575249	7	155,144,702	Intergenic	↑	2.64E-05	
cg26754761	2	177,040,938	Exons	↑	2.65E-05	HOXD3; HAGLR
cg17960141	1	190,141,840	Introns	↑	2.76E-05	BRINP3
cg01423811	2	142,037,701	Introns	↑	2.84E-05	LRP1B
cg25364684	16	53,535,593	Introns	↑	2.84E-05	AKTIP

^a^↑ indicates hypermethylation (higher methylation level in the pain group as compared to the no-pain group), and ↓ indicates hypomethylation (lower methylation level in the pain group as compared to the no-pain group).

^b^Annotated genes within ±5kb of the CpG probe.

**Table 3.** Top 20 differentially methylated regions.

Chr	Start	End	Genomic feature	Direction^a^	# CpG^b^	*p* value	Genes^c^
1	205,818,956	205,819,609	Promoters	↑	12	9.22E-06	PM20D1
5	179,740,743	179,741,120	Exons; introns	↑	4	1.96E-05	GFPT2
2	30,669,597	30,669,863	Promoters	↓	4	3.28E-05	LCLAT1
1	19,110,734	9,111,089	Intergenic	↓	5	5.23E-05	
1	153,599,487	153,599,831	Promoters	↑	11	1.58E-04	S100A13; S100A1
6	30,039,403	30,039,524	Exons; introns	↓	7	2.26E-04	PPP1R11; RNF39
9	36,276,879	36,277,154	Promoters	↓	5	2.33E-04	GNE
14	63,671,231	63,671,737	Promoters	↓	6	2.42E-04	RHOJ
15	101,093,778	101,093,900	Exons	↑	3	2.99E-04	PRKXP1
1	47,900,630	47,900,630	Promoters	↑	1	3.08E-04	FOXD2-AS1; FOXD2
1	42,384,056	42,384,647	Promoters	↓	9	3.24E-04	HIVEP3
11	70,672,835	70,673,256	Introns	↓	7	3.44E-04	SHANK2
14	106,183,770	106,183,770	Introns	↓	1	4.51E-04	
6	30,039,025	30,039,206	Exons	↓	6	4.68E-04	PPP1R11; RNF39
11	66,362,959	66,362,959	Introns	↑	1	4.82E-04	CCDC87; CCS
5	176,797,920	176,798,049	Exons; introns	↓	3	4.92E-04	RGS14
12	9,555,480	9,555,721	Promoters	↑	2	5.14E-04	
1	25,655,526	25,655,526	Exons	↑	1	6.00E-04	RSRP1; RHD
15	30,861,172	30,861,172	Promoters	↓	1	6.39E-04	ULK4P1
6	32,628,305	32,628,305	Introns	↑	1	6.42E-04	HLA-DQB1

^a^↑ indicates hypermethylation (higher methylation level in the pain group as compared to the no-pain group), and ↓ indicates hypomethylation (lower methylation level in the pain group as compared to the no-pain group).

^b^Number of CpG probes within the region.

^c^Annotated genes within ±5kb of the region.

### Genomic distribution of the identified DMPs

**Figure 2.**
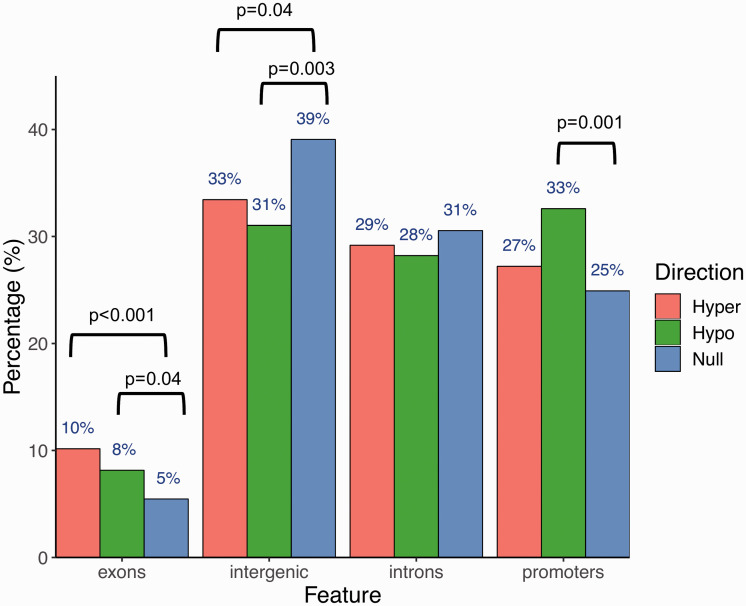
Genomic feature distributions of all putative DMPs (*p* ≤ 0.05).

To examine the potential functional impact of pain-related DMRs on transcriptional activities, we annotated the putative DMRs to predetermined genomic features (Figure 2). Compared to the null distribution of CpG probes included in the Illumina EPIC array, hypermethylated regions were enriched in exons (10% vs. 5%, *p* < 0.001) but depleted in intergenic regions (33% vs. 39%, *p* = 0.04). By contrast, hypomethylated regions were most enriched in promoters (33% vs. 25%, *p* = 0.001), followed by exons (8% vs. 5%, *p* = 0.04), but depleted in intergenic regions (31% vs. 39%, *p* = 0.003).

### Enrichment analysis

**Figure 3.**
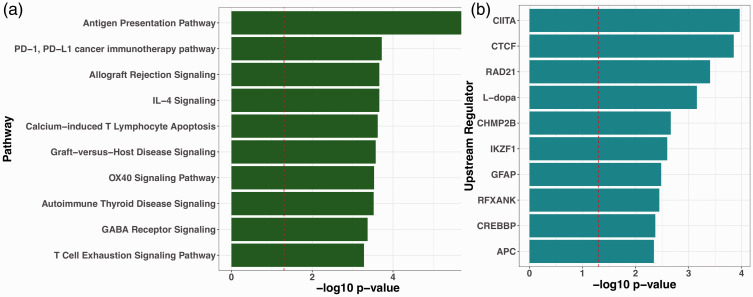
Pathway enrichment analysis using Ingenuity Pathway Analysis: (a) result for top 10 canonical pathways and (b) result for top 10 upstream regulators. The vertical dashed red line indicates the *p* = 0.05 level. PD-1: programed cell death 1 receptor; PD-L1: PD-1 ligand 1; IL-4: interleukin-4; GABA: gamma-aminobutyric acid; APC: antigen-presenting cell.

Pathway enrichment analysis revealed that the pain-related DMRs were enriched across multiple pathways. Figure 3(a) shows the top 10 canonical pathways. Moreover, Figure 3(b) shows the top 10 upstream regulators of these putative DMRs.

### Comethylation networks

We identified 4 comethylated modules from the WGCNA analysis, including the turquoise module (*n* = 43 CpG probes), blue module (*n* = 37), brown module (*n* = 26), and the yellow module (*n* = 21) (see Figure S2). Interestingly, the structure of the blue module in the pain group was not preserved in the no-pain group (Figure S3). The blue module network structure of the pain group is shown in Figure S4, in which the hub CpG was annotated with ATF-2.

## Discussion

In this exploratory study, we evaluated DNA methylation profile associations with self-reported musculoskeletal pain in community-dwelling older adults and employed an integrative computational analysis to identify common, targetable pathways enriched by the genes with differentially methylated CpG sites. We focus our discussion on the top 10 enriched pathways identified, which were reflective of cellular responses important for immune signaling and gamma-aminobutyric acid (GABA) receptor signaling. Further, we discuss the comethylated module hub gene ATF-2 and integrate our findings in relation to pain.

Most of the top 10 pathways identified (i.e. *PD-1/PD-L1 immunotherapy pathway*, *antigen presentation pathway*, *T-lymphocyte apoptosis*, *T cell exhaustion signaling*, *OX40 signaling pathway*, *interleukin-4 (IL-4) signaling*, *autoimmune thyroid disease signaling*) were linked to cellular signaling processes related to T cell activation. T cell activation requires two signals. The first signal involves antigen recognition provided by the interaction of antigenic peptide/major histocompatibility complex (MHC) with the T cell receptor, which confers specificity to the immune response. The second signal is the “costimulatory signal” delivered by costimulatory molecules expressed on antigen-presenting cells to receptors expressed on T cells.^32^ The best studied costimulatory signals are those that include CD28/CD80/B7-1, which contribute to the function of the T effector cells and the CTLA-4/CD86/B7-2, which play a decisive role in maintaining peripheral tolerance and impeding autoimmunity.^33^ On the other hand, the programed cell death 1 receptor (PD-1) has been identified as another inhibitory receptor that is expressed on the surface of activated T cells. Its ligands, PD-1 ligands (PD-Ls), PD-L1 (B7-H1, CD274), and PD-L2 (B7-DC, CD273), are new members of the B7/CD28 family and are expressed on the surface of dendritic cells or macrophages. PD-1/PD-L1, PD-L2 pathway immune checkpoints can result in T cell dysfunction by causing T cell anergy, T cell exhaustion, and T cell apoptosis and by inducing the differentiation of regulatory cells.^34^ Although PD-1/PD-L1 signaling has been mainly targeted for cancer immunotherapy, it may also serve as an endogenous pain inhibitor and a neuromodulator. PD-1 is expressed in nociceptive neurons in the dorsal root ganglion (DRG), and ligand binding to PD-1 triggers hyperpolarization through activation of TREK2 K+ channels.^35^ In animals, PD-L1 interactions with PD-1 has analgesic effects while blockade of either PD-1 or PD-L1 elicits mechanical allodynia. Thus, PD-1/PD-L1 signaling may be a relevant target for future analgesic therapies, consistent with its role in balancing protective immunity and immunopathology to maintain homeostasis.

Tumor necrosis factor receptor superfamily, member 4 (TNFRSF4)/CD134/OX40 is a secondary costimulatory immune checkpoint molecule and its expression is dependent on full activation of the T cell. OX40/OX40L pathway upregulates the antiapoptotic proteins on T cell to increase the cytokine production and memory T cell generation, thus aggravating autoimmune diseases like Graves’ disease, autoimmune arthritis, and uveitis.^33^ In addition, IL-4 signaling is widely involved in various processes such as T cell proliferation, activated B cell stimulation, activation of macrophages, chronic inflammation, and wound repair. IL-4 is mainly produced by activated T cells with a robust literature implicating IL-4 in acute and chronic pain in both animal and human studies. The antinociceptive effect of IL-4 is largely mediated via JAK/STAT activation resulting in the inhibition of the production and/or release of proinflammatory cytokines that indirectly contribute to hyperalgesia by enhancing the synthesis or release of prostaglandins, sympathetic amines, endothelin, and nerve growth factor.^36^ For example, IL-4 deficiency has been described to exacerbate inflammation in collagen-induced arthritis,^37^ and we have previously reported significantly greater IL-4 production in older adults after an experimental pain stimulus compared to a warm control stimulus.^38^ Overall, there is enough evidence supporting a dysregulated anti-inflammatory response that includes IL-4 signaling in acute and chronic pain states, particularly in aging.

The final canonical pathway enriched by genes with DMPs common to pain was the GABA receptor signaling pathway. This finding aligns with previous animal and human literature where pain is associated with GABAergic inhibitory tone in the nervous system.^39,40^ Several basic studies suggest a role of DNA methyltransferases in the regulation of GABAergic gene expression in brain regions relevant for pain including the striatum and hippocampus.^41^ DNA epigenetic modifications of amygdala GABAergic interneurons were involved in anxiety-like behaviors that were reversed with a demethylating agent.^42^ In our own participants with chronic pain, GABA concentrations in the frontal cortex are significantly reduced compared to no-pain controls.^43^ Interestingly, GABA has immunoinhibitory effects on T-cells,^44–46^ and GABA_A_ receptors mediate inhibition of T cell responses.^45^ Emerging research demonstrates that GABAergic activation enhanced antimicrobial responses against intracellular bacterial infection, and in turn, intracellular bacterial infection decreased GABA levels *in vitro* in macrophages and *in vivo* in sera. Further, treatment of macrophages with GABA or GABAergic drugs promoted autophagy activation and enhanced phagosomal maturation and antimicrobial responses. Thus, considering pain exposures as stressful events that can induce exaggerated immune responses in older individuals,^38^ our findings highlight the need to take into consideration relevant neuroimmune interactions and integrate immunology with neuroscience to find novel potential targets for pain.

Finally, the comethylation network module in the pain group that was not preserved in the no-pain group had the hub gene cyclic adenosine monophosphate-dependent transcription factor ATF-2. This transcriptional activator regulates the transcription of various genes, including those involved in antiapoptosis, cell growth, and DNA damage response. In the nucleus, it contributes to global transcription and the DNA damage response, in addition to specific transcriptional activities that are related to cell development, proliferation, and death. In the cytoplasm, it impairs mitochondrial membrane potential, inducing mitochondrial leakage and promoting cell death. ATF-2 signal transduction pathways were activated in a rat model of inflammatory pain that was reversed after treatment suggesting an active role for ATF-2 in regulating inflammatory pain.^47^

Our study has several limitations worth considering. First, our study sample size was very small, not allowing global adjustments for multiple comparisons, which increases the risk of false-positive results. Second, our analysis was based on whole blood samples and not specific nervous system tissue important for pain processing (e.g. brain, DRG). Given that routine invasive collection of central nervous system tissues in humans is not feasible, research using blood samples is imperative to move the field forward. Nonetheless, previous research^48^ suggests a high correlation between brain tissue and blood methylation patterns. Third, variations in blood cell composition may affect the results of the methylation analysis, although in other pain studies this was not observed.^49^ Analysis of whole blood^50,51^ and lymphocyte^52,53^ samples from individuals exposed to various forms of early-life adversity has consistently revealed aberrant methylation patterns that are present on a genome-wide scale. Peripheral cells such as lymphocytes also offer an avenue to examine the hypothalamic–pituitary axis (HPA), as lymphocytes are sensitive to HPA endocrine modulation.^38,54^ Fourth, it is not currently known whether the observed epigenetic patterns are a cause or a consequence of chronic pain in our participants. Moreover, although we screened our participants over the phone and again in person during a medical interview to exclude conditions that may confound our results, it is still possible that some of the above epigenetic markers were picking up early cellular signaling changes associated with cancer or other medical conditions associated with aging, unbeknownst to our participants. This is plausible since age is the major risk factor for cancer development. Another example is the intriguing signaling of allograft rejection and graft-versus-host disease, which is mainly involved in immune responses after organ transplantation, also not reported in the medical interviews by our participants. Finally, we used computational analyses to evaluate the pathways associated with epigenetic group differences but did not examine genetic or measure protein expression levels. Given the complexity and multiple levels of gene regulation, future larger studies are needed to evaluate not only gene regulation using epigenetics but actual gene and protein expression levels in relation to pain. However, previous studies have reported MHC class I and class II immune-related genes to be associated with chronic pain phenotypes,^55–57^ including a recent study implicating immune signaling in the transition from acute to chronic pain in persons with low-back pain.^58^ Therefore, our findings need to be urgently replicated in larger studies to address these limitations.

Despite the above caveats, our study provides preliminary insight into potential mechanistic changes, at the cellular level, that are associated with chronic musculoskeletal pain in older individuals. Cellular signaling pathways regulate everything in the life of a cell including responding to stress, protecting itself from harm (e.g. environmental insults or infections), as well as death by apoptosis. These signaling pathways are important for various aspects of the immune responses and overall system functioning. Although preliminary in nature, our study is consistent with previous studies in other pain conditions^17,48,49,59^ and also provides additional areas worthy of further study. For example, we found differential methylation in genomic distribution location (e.g. promoters, introns), and while methylation in gene promoters is generally associated with transcriptional silencing, methylation of the first intron is linked with gene expression.^60^ The field of epigenetics can move forward our understanding of complex behaviors such as the pain experience from simple individual contributors to global and multiple layers of regulatory cues along multiple levels of the neural axis. Future larger human studies in well-characterized cohorts are needed to integrate multilayer epigenomic data, together with genomic, transcriptomic, and proteomic data to comprehend how epigenetic information contributes to complex regulatory processes involved in chronic pain in aging.

## Supplemental Material

sj-pdf-1-mpx-10.1177_1744806920966902 - Supplemental material for Enrichment of genomic pathways based on differential DNA methylation profiles associated with chronic musculoskeletal pain in older adults: An exploratory study Click here for additional data file. Supplemental material, sj-pdf-1-mpx-10.1177_1744806920966902 for Enrichment of genomic pathways based on differential DNA methylation profiles associated with chronic musculoskeletal pain in older adults: An exploratory study by Soamy Montesino-Goicolea, Puja Sinha, Zhiguang Huo, Asha Rani, Thomas C Foster and Yenisel Cruz-Almeida in Molecular Pain

sj-xls-2-mpx-10.1177_1744806920966902 - Supplemental material for Enrichment of genomic pathways based on differential DNA methylation profiles associated with chronic musculoskeletal pain in older adults: An exploratory study Click here for additional data file. Supplemental material, sj-xls-2-mpx-10.1177_1744806920966902 for Enrichment of genomic pathways based on differential DNA methylation profiles associated with chronic musculoskeletal pain in older adults: An exploratory study by Soamy Montesino-Goicolea, Puja Sinha, Zhiguang Huo, Asha Rani, Thomas C Foster and Yenisel Cruz-Almeida in Molecular Pain

sj-xls-3-mpx-10.1177_1744806920966902 - Supplemental material for Enrichment of genomic pathways based on differential DNA methylation profiles associated with chronic musculoskeletal pain in older adults: An exploratory study Click here for additional data file. Supplemental material, sj-xls-3-mpx-10.1177_1744806920966902 for Enrichment of genomic pathways based on differential DNA methylation profiles associated with chronic musculoskeletal pain in older adults: An exploratory study by Soamy Montesino-Goicolea, Puja Sinha, Zhiguang Huo, Asha Rani, Thomas C Foster and Yenisel Cruz-Almeida in Molecular Pain
