# Supplementary material for: Enrichment of genomic pathways based on differential DNA methylation profiles associated with chronic musculoskeletal pain in older adults: An exploratory study
Source: Mol Pain. 2020 Oct 18;16:1744806920966902. doi: 10.1177/1744806920966902 (PMC7711149; doi:10.1177/1744806920966902)
Supplement: sj-pdf-1-mpx-10.1177_1744806920966902 - Supplemental material for Enrichment of genomic pathways based on differential DNA methylation profiles associated with chronic musculoskeletal pain in older adults: An exploratory study [file sj-pdf-1-mpx-10.1177_1744806920966902.pdf]

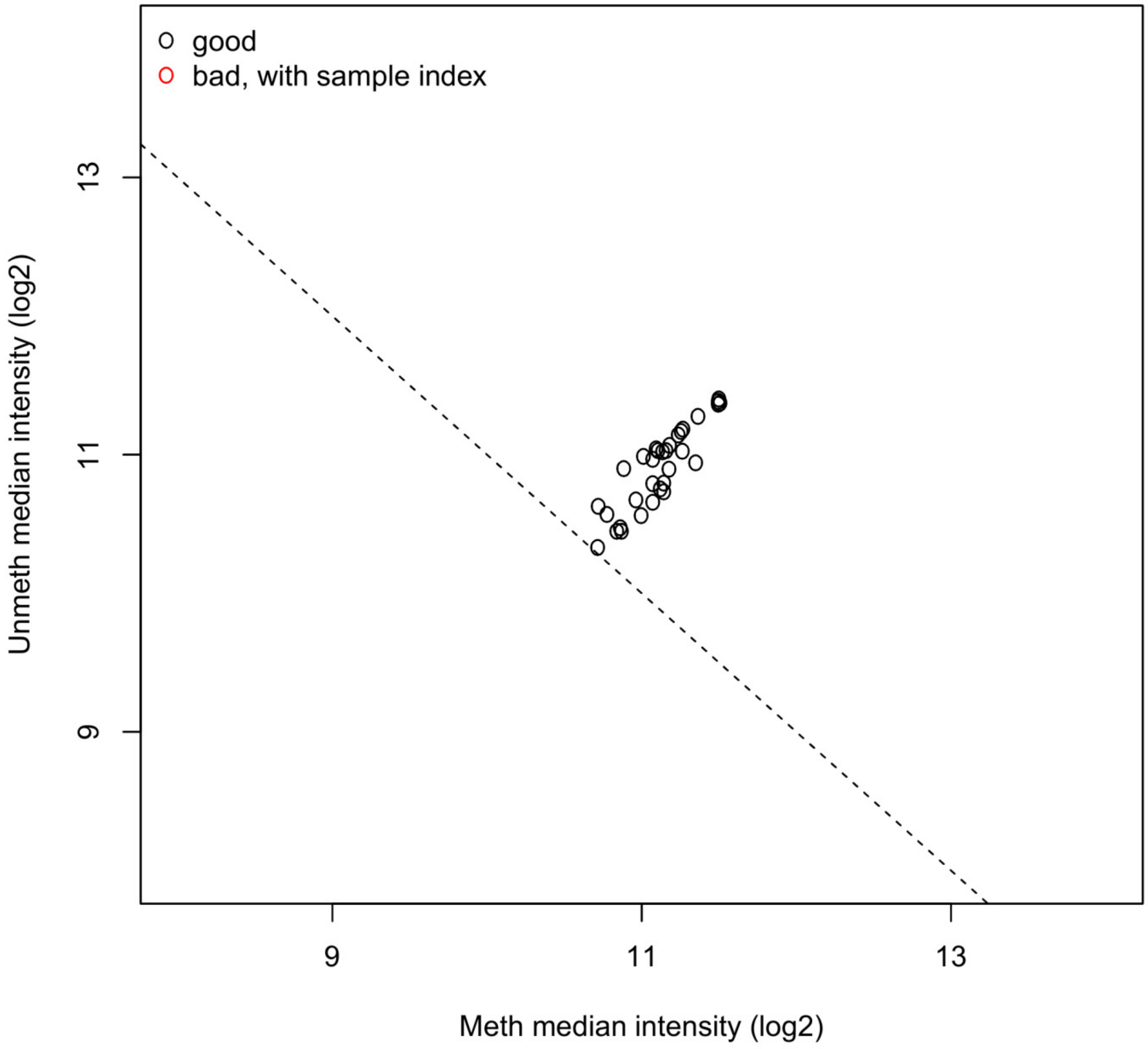

Figure S1. Quality control of DNA methylation samples.

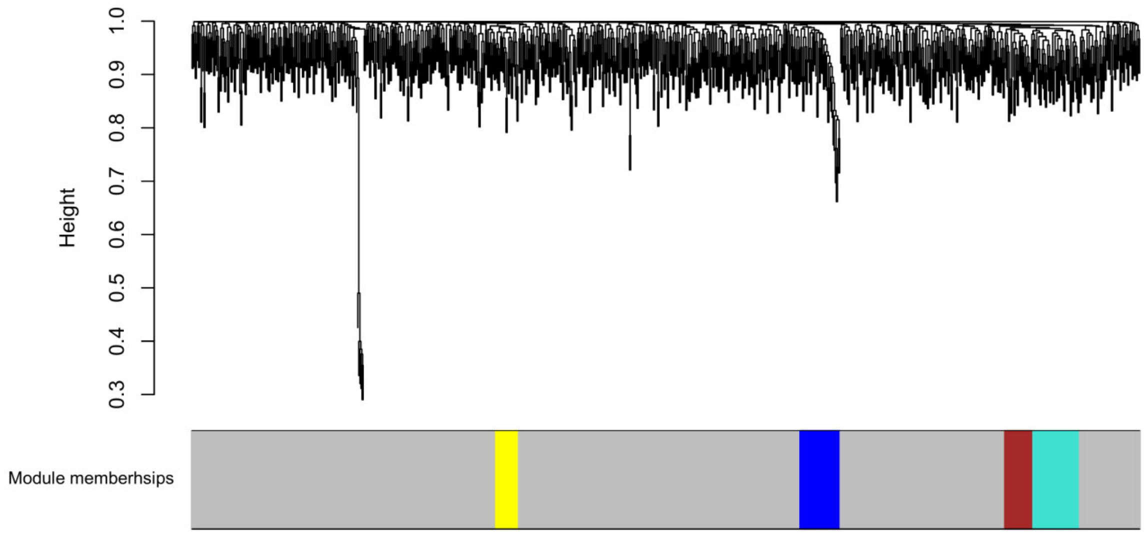

Figure S2. Hierarchical clustering analysis for the 855 putative pain-related DMPs ($p<0.001$). Co-methylated modules were identified by weighted gene co-expression network analysis (WGCNA). Co-regulation dendrogram were obtained by clustering the dissimilarity based on topological overlap matrix distance. A total of 4 modules were identified, with each module represented by an arbitrary color.

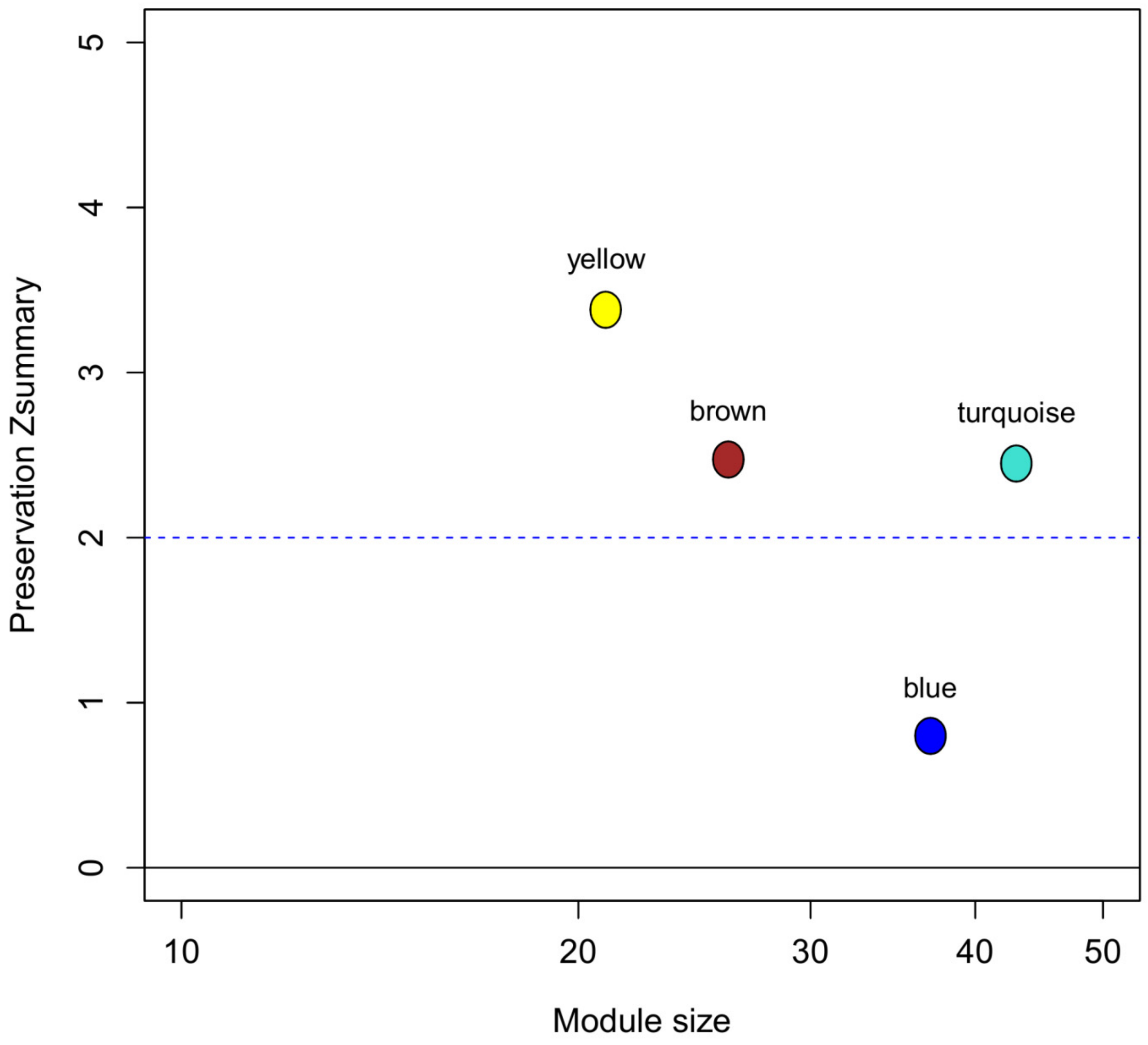

Figure S3. Module preservation analyses. Module below the blue dashed line indicates the structure of the module in the pain group is not preserved in the no-pain group

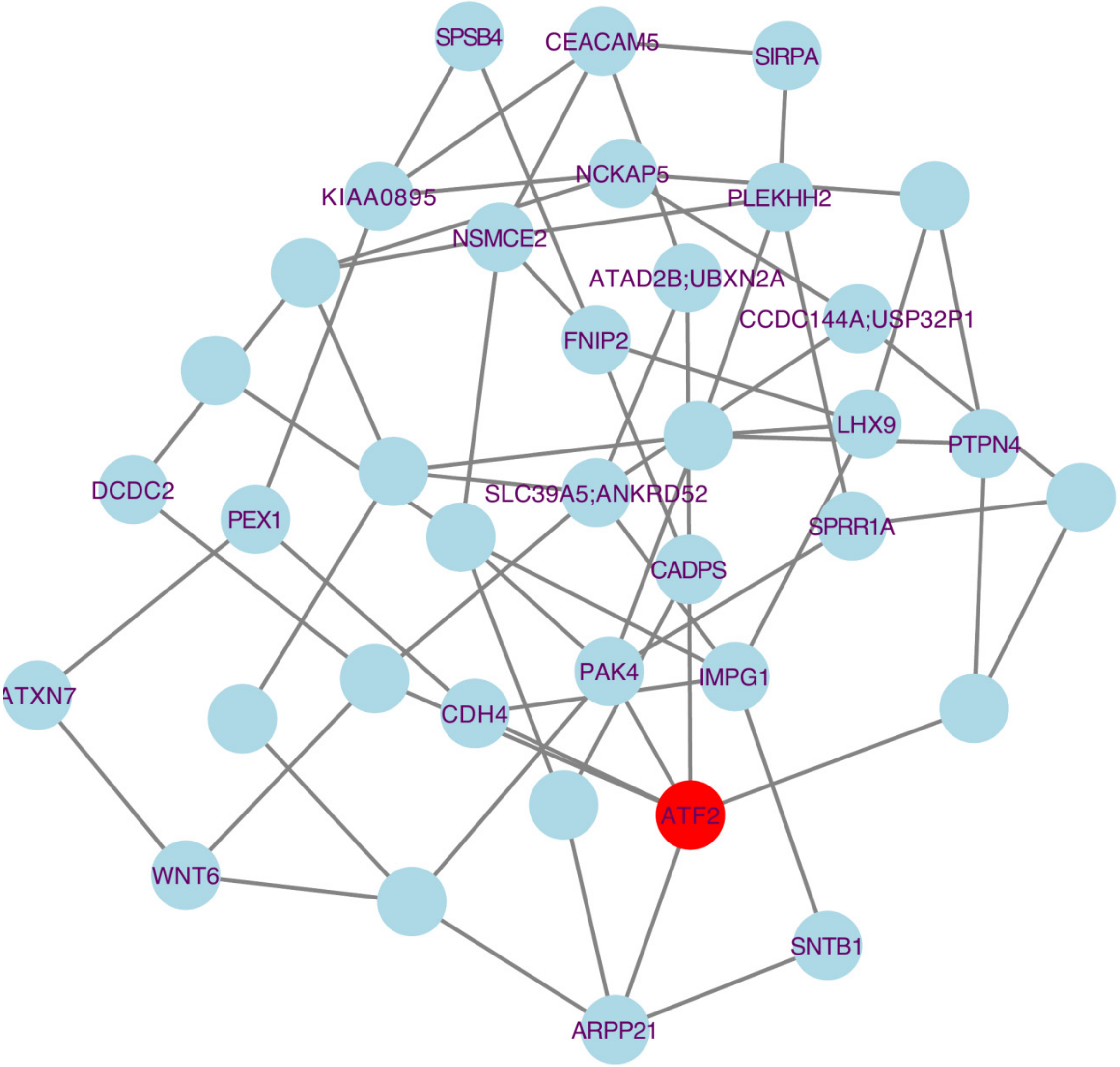

Figure S4. Blue module structure of the pain group. Red color node indicates the hub gene.
